# Cross-omics association of small extracellular vesicle miRNAs and metabolites in seminal plasma of idiopathic male infertility

**DOI:** 10.3389/fendo.2026.1935941

**Published:** 2026-09-09

**Authors:** Chao Du, Xiaoyue Zhai, Xinyue Fan, Ao Li, Lisha Chen, Mingyang Gao, Yining An, Yuexin Yu

**Affiliations:** 1 Department of Reproductive Medicine, General Hospital of Northern Theater Command, Shenyang, Liaoning, China; 2 Department of Histology and Embryology, School of Basic Medicine, China Medical University, Shenyang, Liaoning, China; 3 Student Affairs Department of Shengjing Hospital, China Medical University, Shenyang, Liaoning, China; 4 School of Graduate Studies, China Medical University, Shenyang, Liaoning, China

**Keywords:** extracellular vesicles, idiopathic male infertility, microRNAs, oxidative stress, phospholipase D signaling pathway, seminal plasma, untargeted metabolomics

## Abstract

**Background:**

Idiopathic male infertility (IMI) accounts for 30%–40% of male infertility cases, yet the molecular networks associated with seminal plasma dysfunction and non-invasive biomarkers remain largely unclear. This study aimed to preliminarily explore the transcriptomic and metabolomic alterations in IMI seminal plasma through cross-omics integration, and to screen for exploratory candidate discriminators among the metabolites.

**Methods:**

A total of 30 IMI patients and 30 normozoospermic controls were recruited for untargeted UHPLC-MS/MS metabolomics of seminal plasma. Candidate differential metabolites were screened and subjected to KEGG and Gene Set Enrichment Analysis (GSEA); ROC analysis with bootstrap internal validation was conducted on candidate differential metabolites to explore candidate discriminators. For miRNA sequencing, 10 subjects were randomly selected from each group to isolate and identify seminal plasma small extracellular vesicles (sp-sEVs) following International Society for Extracellular Vesicles (ISEV) standards. Subsequent bioinformatic analyses included miRNA differential expression screening, target gene prediction, GO/KEGG enrichment, protein–protein interaction (PPI) network construction, and pairwise Pearson correlation analysis between dysregulated miRNAs and differential metabolites within the nested subgroup.

**Results:**

We identified 68 and 152 candidate differential metabolites under negative and positive electrospray ionization modes, respectively. Metabolite perturbations were predominantly enriched in unsaturated fatty acid biosynthesis, whereas the arginine and proline metabolism pathway was globally suppressed in IMI patients. The phosphatidic acid derivative PA(i‑15:0/18:1(12Z)‑2OH(9,10)) showed the best discriminatory potential among all screened metabolites, with a AUC of 0.822 (95% CI: 0.716–0.929). miRNA sequencing uncovered 128 differentially expressed miRNAs (62 upregulated, 66 downregulated) in IMI sp-sEVs. Functional enrichment of miRNA target genes highlighted perturbed protein phosphorylation and intracellular signal transduction, with the phospholipase D (PLD) signaling pathway as the most statistically significant pathway; *PIK3CA*, *PIK3R1*, and *AKT1* were suggested to be core hub genes within the PLD network. Correlation analysis suggested that upregulated miRNAs (hsa-miR-141-5p, hsa-miR-28-5p) were positively correlated with up-regulated N-cis-11,14-eicosadienoyl ethanolamine in seminal plasma.

**Conclusion:**

Coordinated alterations between sp-sEV miRNAs and seminal plasma metabolites were observed in IMI. Several candidate metabolites showed discriminatory value in this single cohort, but require validation. The PLD pathway may be involved, though causality remains unconfirmed.

## 1 Introduction

The World Health Organization defines infertility as the failure to achieve a clinical pregnancy after 12 months or more of regular unprotected sexual intercourse (1). Infertility has become a major global health burden (2, 3), affecting approximately 187 million couples worldwide, with male factors contributing partially or entirely to about 50% of cases (4, 5). Among infertile men, approximately 30%–40% of patients still cannot be clearly diagnosed despite routine clinical evaluations (including reproductive tract infection screening, endocrine hormone testing, and genetic testing); these cases are classified as idiopathic male infertility (IMI) (6).

Numerous studies have confirmed that idiopathic infertility may be associated with dietary intake (7), environmental factors (8), comorbidities (9) and genetic factors (10, 11). Seminal plasma, a critical component of semen, is composed of secretions from the accessory sex glands (seminal vesicles, prostate, and bulbourethral glands), the epididymis, and other tissues, accounting for approximately 95% of semen volume (12). Seminal plasma serves as the direct microenvironment for sperm maturation, energy supply, and motility regulation. The small-molecule metabolites contained within it not only accurately reflect the secretory functions of the accessory glands and epididymis, but also directly participate in ATP energy production for sperm motility, maintenance of the antioxidant defense system, and stabilization of sperm membrane lipid composition (13). In recent years, untargeted metabolomics, owing to its high-throughput and unbiased nature, has demonstrated unique advantages in screening biomarkers and uncovering pathological pathways in infertility (14). For example, Xu S et al. found that acylcarnitine levels were positively correlated with semen quality and sperm concentration (15); Wang YX et al., using untargeted metabolomic sequencing, reported that elevated urinary metabolites of phthalates might affect semen quality by inducing disturbances in polyunsaturated fatty acids and acylcarnitine metabolism in seminal plasma (16). Collectively, these studies indicate that aberrant seminal plasma metabolites represent important molecular features of impaired spermatogenesis and dysfunction; however, metabolic changes are often driven by upstream regulatory signaling networks.

Beyond small-molecule metabolites, seminal plasma is also rich in small extracellular vesicles (sEVs)-membrane-bound vesicles with diameters of 30–150 nm secreted by reproductive tract epithelial cells, which carry abundant bioactive molecules including miRNAs, proteins, and lipids (17). Recent evidence has shown that seminal plasma small extracellular vesicles (sp-sEVs) can regulate reproductive system functions by delivering their cargo to target cells, such as enhancing sperm motility, promoting sperm maturation, and modulating sperm movement (18, 19). Notably, patients with abnormal semen quality exhibit significant alterations in the miRNA expression profiles of sp-sEVs. For instance, Eikmans et al. reported that miR-34b-5p levels were significantly elevated in normal controls compared with the asthenozoospermia group (20); in another study, compared with normozoospermic men, men with oligoasthenozoospermia and subfertility showed significantly elevated expression of miR-765 and miR-1275, and significantly decreased expression of miR-15a (21). These findings suggest that dysregulated post-transcriptional regulation mediated by sEV-derived miRNAs may be a key upstream trigger for seminal plasma microenvironment disturbances and impaired sperm motility.

Nevertheless, there is still a lack of systematic integrative analysis of the intrinsic associations between the miRNA transcriptional regulatory network and metabolic pathways. In light of this, the present study designed a nested multi-omics integration strategy based on overlapping samples. First, we enrolled 30 IMI patients and 30 normozoospermic controls, and performed untargeted metabolomics on seminal plasma to systematically delineate the disease-associated metabolite profile and key perturbed pathways. Subsequently, we randomly selected 10 subjects from each group as a subgroup for sp-sEVs extraction, identification, and miRNA transcriptome sequencing. Finally, we explored the expression concordance between sEV-derived miRNAs and metabolites via correlation analysis. This study aims to uncover novel mechanisms of idiopathic male infertility from a synergistic perspective of miRNA–target gene–metabolite interactions, and to provide multi-omics evidence for biomarker discovery and targeted therapy of this condition.

## 2 Subjects and methods

### 2.1 Study subjects

A total of 30 patients with idiopathic male infertility (IMI group) and 30 normozoospermic controls (CG group) were enrolled in this study. All participants underwent routine semen analysis and untargeted metabolomics profiling of seminal plasma, constituting the metabolomics cohort (n = 60). From this cohort, 10 patients and 10 controls were randomly selected for additional sEVs extraction and miRNA transcriptome sequencing. Semen analysis and interpretation were strictly performed in accordance with the criteria of the *WHO laboratory manual for the examination and processing of human semen* (5th edition, 2010) (22): asthenozoospermia was defined as progressive motility (PR) < 32%; oligozoospermia as sperm concentration < 15 × 10^6^/mL; and teratozoospermia as normal morphology rate < 4%. Patients in the case group could present with a single abnormality in semen parameters or a combination of two or more abnormalities.

Prior to study enrollment, all potential confounding factors affecting male fertility were strictly excluded according to unified screening criteria. Baseline information including infertility duration was recorded for each infertile couple; Males with recent fever, systemic metabolic diseases, ongoing long-term medication, regular antioxidant intake and persistent harmful environmental exposure were excluded after medical history inquiry; varicocele was excluded by physical examination combined with scrotal ultrasound; leukocytospermia was ruled out based on routine semen testing. All female partners received complete gynecological assessment to eliminate any female-factor infertility (including ovarian dysfunction, tubal lesions, endometriosis and other gynecological infertility diseases).

This study was conducted in accordance with the Declaration of Helsinki and was approved by the Ethics Committee of General Hospital of Northern Theater Command (Approval No. 2025-03). All participants provided written informed consent.

### 2.2 Sample collection

Semen samples were collected by masturbation after 2–7 days of abstinence. Routine semen analysis was performed according to the World Health Organization (WHO) guidelines, including assessment of semen volume, sperm concentration, progressive motility, sperm DNA fragmentation index (DFI), High DNA stainability (HDS) and morphology. The remaining semen samples were centrifuged at 3,000 × g for 10 minutes at 4°C, aliquoted into RNase-free Eppendorf tubes, and snap-frozen in liquid nitrogen.

### 2.3 Untargeted metabolomics of seminal plasma

#### 2.3.1 Sample preparation and UHPLC-MS/MS analysis

An aliquot of 100 μL seminal plasma was mixed with 300 μL pre-cooled 80% aqueous methanol, vortexed, and incubated on ice for 5 minutes, followed by centrifugation at 15,000 × g for 20 minutes at 4°C. An appropriate volume of the supernatant was diluted with LC-MS grade ultrapure water to a final methanol concentration of 53%, and centrifuged again at 15,000 × g for 20 minutes at 4°C. The final supernatant was collected for injection. An equal volume of supernatant from each sample was pooled to prepare quality control (QC) samples, which were used to monitor instrument stability and data reproducibility.

Chromatographic separation was performed on a Vanquish UHPLC system (Thermo Fisher, Germany) coupled to an Orbitrap Q Exactive™ HF mass spectrometer (Thermo Fisher, Germany). The analytical column was an ACQUITY UPLC BEH Amide column (100 mm × 2.1 mm, 1.7 μm). The mobile phases consisted of (A) 90% acetonitrile in water containing 5 mM ammonium acetate and (B) 50% acetonitrile in water containing 5 mM ammonium acetate. The flow rate was 0.2 mL/min, and the gradient elution program ran for 12 minutes. Mass spectrometry was performed using an electrospray ionization (ESI) source in both positive and negative ion modes. The spray voltage was set at 3.5 kV, capillary temperature at 320°C, and the mass scan range was m/z 70–1,050.

#### 2.3.2 Data processing and differential metabolite screening

Raw data were processed using XCMS software (23) for peak alignment, peak detection, and peak area quantification. Metabolite identification was performed based on accurate molecular mass (mass tolerance ≤ 10 ppm) by matching against the NovoMetDB database. Metabolites with > 50% missing values were excluded, and the remaining missing values were imputed using the K-nearest neighbor (KNN) algorithm (24). Compounds with a coefficient of variation (CV) > 30% in QC samples were removed (25). Metabolite annotation was performed using the HMDB database (https://hmdb.ca/metabolites).

Partial least squares discriminant analysis (PLS-DA) was conducted using the metaX software package (26). Prior to statistical testing, raw metabolite abundance values were subjected to natural logarithmic transformation to normalize skewed intensity distributions. Welch’s corrected independent samples t-test was performed on log-transformed data to calculate raw *P-values*. Candidate differential metabolites were preliminarily selected based on the combined criteria of variable importance in projection (VIP) >1, *P* < 0.05, and |log2(fold change)| ≥ 0.26. The identified differential metabolites were then used as the input set for KEGG pathway enrichment analysis using the hypergeometric distribution algorithm, with a significance threshold of *P* < 0.05. The Benjamini-Hochberg FDR correction was applied to control the false discovery rate. Both raw *P* values and *adjusted P-value (P.adj)* were reported. To avoid information loss caused by the cutoff thresholds in conventional differential screening, Gene Set Enrichment Analysis (GSEA) (27) was further performed. All metabolites were ranked by their fold changes between groups, with KEGG metabolic pathways as predefined gene sets. The permutation number was set to 1,000, and pathways with a normalized enrichment score (NES) and *P.adj* < 0.05 were considered significantly up- or down-regulated in overall abundance.

Receiver operating characteristic (ROC) curve analysis was performed using differential metabolites with significant correlations to IMI to preliminarily screen exploratory candidate discriminants within the present single cohort. The area under the curve (AUC), 95% confidence interval (95% CI), optimal cut-off value, corresponding sensitivity and specificity of each metabolite were calculated and recorded. Bootstrap resampling (1000 iterations) was adopted for internal validation to assess the stability of the exploratory discriminatory performance and to provide bias-corrected estimates of the AUC, thereby reducing the potential over-optimism inherent in single-cohort discovery analyses.

### 2.4 Isolation, characterization, and miRNA sequencing of sp-sEVs

#### 2.4.1 sp-sEVs isolation

Seminal plasma samples were rapidly thawed in a 37°C water bath. The samples were sequentially centrifuged at 2,000 × g for 30 minutes at 4°C to remove cell debris, and then at 10,000 × g for 45 minutes at 4°C to remove larger vesicles. The supernatant was filtered through a 0.45 μm filter to remove residual debris. The filtrate was collected and ultracentrifuged at 100,000 × g for 90 minutes at 4°C. After discarding the supernatant, the pellet was resuspended in 10 mL of pre-cooled PBS and ultracentrifuged again at 100,000 × g for 90 minutes at 4°C. The supernatant was removed, and the sEVs pellet was resuspended in 200 μL of pre-cooled PBS, aliquoted, and stored at −80°C until further use.

#### 2.4.2 Characterization of sp-sEVs

To verify the isolation quality, one representative sample from each group was characterized following ISEV guidelines: (1) Transmission electron microscopy (TEM): 10 μL of the sEVs suspension was applied onto a copper grid and allowed to settle for 1 minute. After removing the floating liquid with filter paper, the grid was negatively stained with 10 μL of 2% uranyl acetate for 1 minute, air-dried, and then observed and imaged under a Hitachi HT-7700 transmission electron microscope at 80 kV. (2) Nanoparticle size analysis: 10 μL of sEV sample was diluted to an appropriate concentration, and the particle size distribution and particle concentration were measured using a NanoFCM N30E nanoparticle analyzer. (3) Western blot detection of marker proteins: sEV suspension was mixed with 5× RIPA lysis buffer and lysed on ice for 30 minutes. Protein concentration was determined using the BCA method. Equal amounts of protein were separated by 12% SDS-PAGE and transferred onto PVDF membranes. After blocking with 5% non-fat milk for 1 hour, the membranes were incubated overnight at 4 °C with primary antibodies against CD9 (Boster, 1:1,000), TSG101 (Abcam, 1:1,000), and the negative control anti-Calnexin (SAB, 1:1,000). After washing, the membranes were incubated with HRP-conjugated goat anti-rabbit secondary antibody (Invitrogen, 1:5,000) for 1 hour at room temperature, and signals were visualized using an ECL chemiluminescence system.

#### 2.4.3 miRNA sequencing and differential expression analysis

sEV-derived miRNA was extracted using the miRNeasy Serum/Plasma Kit (QIAGEN). Libraries were constructed using the VAHTS Small RNA Library Prep Kit for Illumina V2 (NR811-C4, Vazyme) and sequenced on an Illumina NovaSeq X Plus platform with single-end 50-bp reads (SE50), achieving a sequencing depth of ≥10 million raw reads per sample. Raw reads were processed using FastQC for quality assessment, followed by adapter trimming and low-quality read filtering using cutadapt. The clean reads were then aligned against the GRCh38 reference genome using Bowtie (28). miRDeep2 combined with srna-tools-cli was used to characterize sRNA profiles for each sample, including secondary structures, sequences, lengths, and read counts of known miRNAs. Known and novel miRNAs were identified collaboratively by miREvo and miRDeep2 software (29). Differential expression analysis of miRNAs was performed using DESeq2 based on raw read counts, with the threshold set at |log_2_FC| > 0 and *adjusted P-value (P.adj)* < 0.05. The expression levels of miRNAs were normalized and calculated as transcripts per million (TPM).

### 2.5 Bioinformatics and multi-omics integrative analysis

#### 2.5.1 miRNA target gene prediction and enrichment analysis

Target genes of differentially expressed miRNAs were predicted using the miRDB database. GO functional enrichment and KEGG pathway enrichment analyses were performed using clusterProfiler (v4.0), with a significance threshold of *P* < 0.05. Protein–protein interaction (PPI) networks of target genes enriched in the core pathways were constructed using Cytoscape (v3.10.2) with the cytoHubba plugin (30, 31) to screen for hub target genes.

#### 2.5.2 miRNA–metabolite correlation analysis

Pearson correlation analysis was separately performed within the IMI subgroup (n = 10) and normozoospermic control (CG) subgroup (n = 10) to quantify linear correlations between top 10 significantly dysregulated sp-sEV miRNAs and top 10 candidate differential metabolites screened from ESI^-^ and ESI^+^ modes. Two filtering criteria were set to retain robust association pairs: absolute correlation coefficient |r| > 0.6 and unadjusted *P* < 0.05. To address multiple testing bias from pairwise multi-omics screening, both raw *P*-values and *P.adj* (Benjamini–Hochberg correction) were summarized in **Supplementary Tables**.

### 2.6 Statistical analysis

Data normality was assessed using the Shapiro–Wilk test, and homogeneity of variances was evaluated using Levene’s test. For continuous variables that followed a normal distribution and had homogeneous variances, data were expressed as mean ± standard deviation ( $\overset{—}{x}$ ± s), and comparisons between the two groups were performed using the independent samples t-test. For variables that did not follow a normal distribution, data were expressed as median (interquartile range) [M (P25, P75)], and comparisons between the two groups were performed using the Mann–Whitney U test. Categorical variables were expressed as counts (percentages), and comparisons between groups were performed using the chi-square test. All statistical analyses were performed using SPSS (v26.0) and R (v4.1.0), with *P* < 0.05 considered statistically significant.

## 3 Results

### 3.1 General characteristics and semen parameters of the two groups

The average infertility duration of the IMI group was 3.07 years. No statistically significant differences were observed between the two groups in terms of age, body mass index (BMI), smoking status, sex hormones [testosterone (T), follicle-stimulating hormone (FSH), luteinizing hormone (LH), and estradiol (E2)], or testicular volume (*P* > 0.05). Compared with the CG group, the IMI group exhibited significantly higher semen volume, DFI and HDS, while sperm concentration, sperm motility, and normal morphology rate were significantly lower (*P* < 0.05). Detailed parameters for each group are presented in **Table 1**. All patients in the IMI group exhibited asthenozoospermia including 8 cases of isolated asthenozoospermia, 1 case complicated with oligozoospermia, 16 cases complicated with teratozoospermia, and 5 cases of oligoasthenoteratozoospermia. Individual clinical information of all participants is summarized in **Supplementary Table 1**.

**Table 1:** Comparison of clinical information and semen parameters between the two groups.

Characteristics	CG group (n=30)	IMI group (n=30)	*t/U/χ²*	*P*
Age (year)	31.00 (29.25, 34.50)	33.00 (31.00, 34.00)	576.500	0.0608
BMI (kg/m²)	25.95 (24.68, 27.70)	24.70 (22.88, 27.50)	338.000	0.0991
Smoke	10/30 (33.3%)	6/30 (20%)	0.767	0.3811
Duration of abstinence (day)	4.00 (3.00, 5.00)	4.00 (3.00, 5.75)	455.000	0.9456
Semen volume (mL)	3.16 ± 1.11	4.06 ± 1.80	-2.338	0.0236
Sperm concentration (×10^6^/mL)	51.33 (32.08, 61.52)	25.86 (16.59, 52.30)	304.500	0.0320
Progressive motility (%)	55.28 ± 15.42	19.59 ± 8.51	11.0976	<0.001
normal spermatozoa (%)	4.00 (4.00, 5.00)	2.00 (1.00, 4.00)	156.000	<0.001
DFI (%)	4.55 (3.03, 6.52)	10.88 (5.76, 14.64)	724.500	<0.001
HDS (%)	6.04 (4.54, 8.02)	9.64 (7.92, 12.07)	701.000	<0.001
T (ng/mL)	3.79 (3.33,4.53)	4.08 (3.42,4.60)	485.000	0.610
LH (IU/L)	4.32 ± 1.37	4.63 ± 1.73	-0.7660	0.447
FSH (IU/L)	3.64 (2.83,5.00)	4.35 (3.17,4.97)	478.000	0.684
E2 (pg/mL)	32.06 ± 8.85	33.10 ± 7.91	-0.479	0.634
Mean testicular volume (mL)	14.83 (13.29,16.97)	15.11 (13.12,16.35)	437.000	0.853

### 3.2 Untargeted metabolomics analysis of seminal plasma

#### 3.2.1 Data quality control

**Figure 1:**
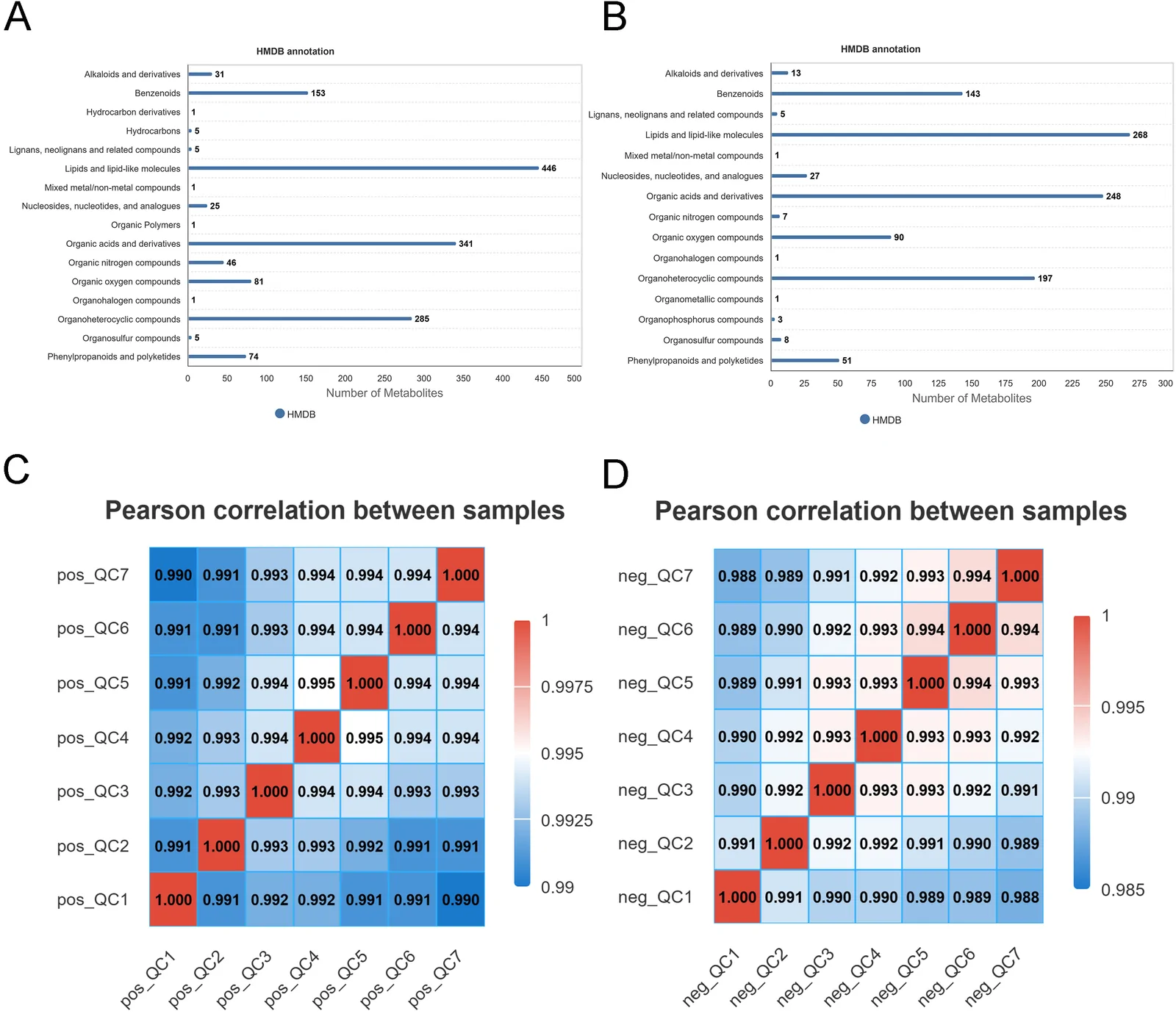
Quality control and metabolite annotation of untargeted metabolomics in seminal plasma. **(A, B)** HMDB chemical taxonomy classification of annotated metabolites in positive (ESI+) and negative (ESI−) ionization modes, showing the number and proportion of metabolites in each category. **(C, D)** Pearson correlation matrices of quality control (QC) samples interspersed throughout the analytical sequence in ESI+ and ESI− modes, respectively.

Untargeted metabolomics detection of all seminal plasma samples was performed in both positive (ESI+) and negative (ESI−) electrospray ionization modes. A total of 2,959 metabolic features were detected in ESI+ mode and 2,123 in ESI− mode. The annotated metabolites from both ionization modes were classified by chemical taxonomy based on the HMDB database (**Figures 1A, B**). In both ESI+ and ESI− modes, the top three categories by abundance were lipids and lipid-like molecules (ESI+: 446; ESI−: 268), organic acids and derivatives (ESI+: 341; ESI−: 248), and organoheterocyclic compounds (ESI+: 285; ESI−: 197). To evaluate the stability of mass spectrometry detection, Pearson correlation analysis was performed on the pooled QC samples interspersed throughout the sequence (**Figures 1C, D**). The results showed that the correlation coefficients of QC samples in both ESI+ and ESI− modes approached 1, indicating good instrument reproducibility and stable, reliable data. Detailed annotation parameters for all metabolites (including retention time, precursor ion m/z, adduct form, mass error, spectral match score, and identification confidence level) are provided in **Supplementary Tables 2.1** and **S2.2**.

#### 3.2.2 Candidate differential metabolite analysis

**Figure 2:**
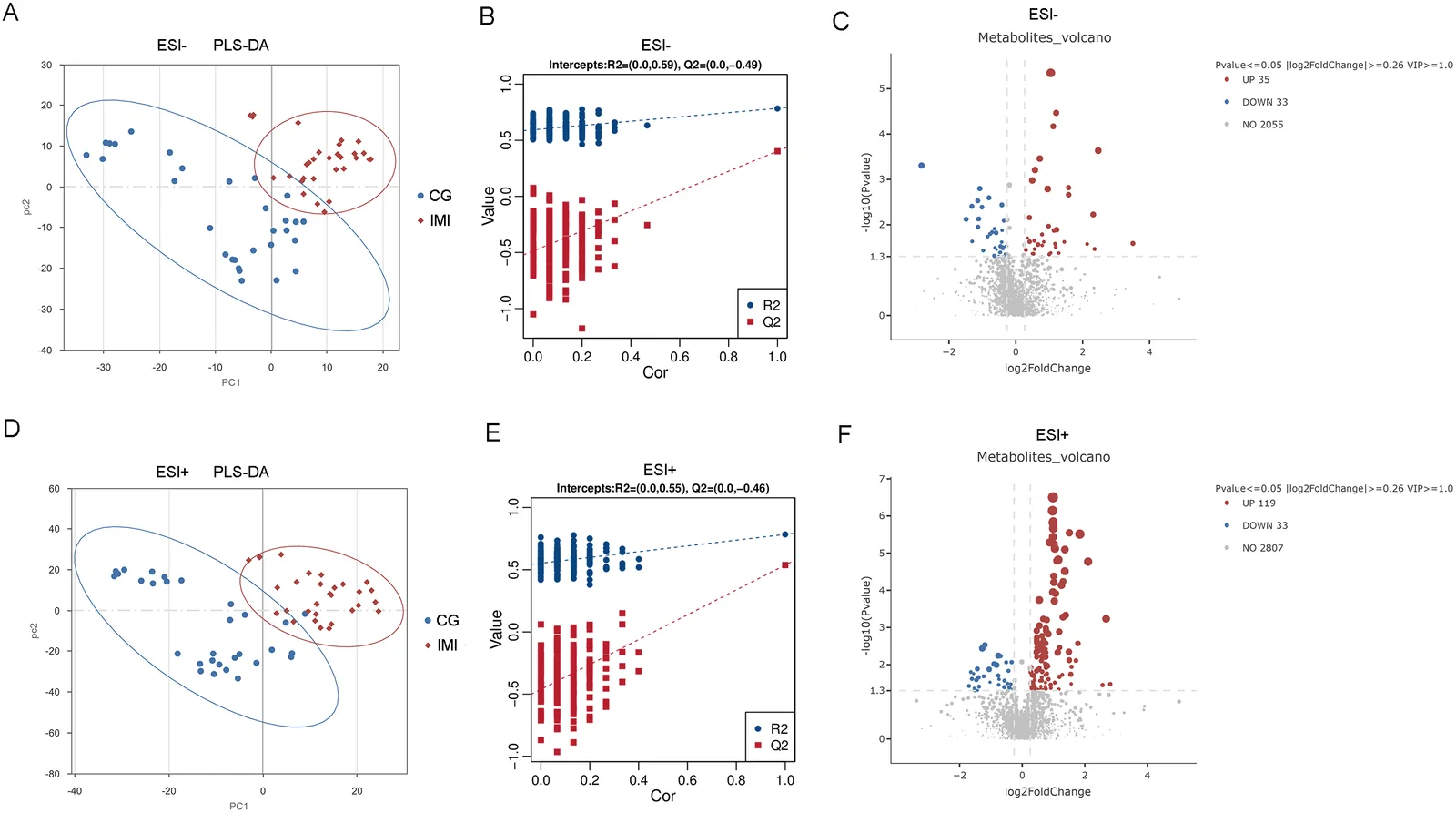
Multivariate analysis and differential metabolite screening in seminal plasma between IMI and CG groups. **(A, D)** PLS-DA score plots showing the separation between IMI and CG groups in ESI− and ESI+ modes, respectively. **(B, E)** Permutation tests (200 random permutations) for the PLS-DA models in ESI− and ESI+ modes, with Q² intercepts of −0.49 and −0.46, respectively, indicating no overfitting. **(C, F)** Volcano plots of candidate differential metabolites in ESI− and ESI+ modes. Red and blue dots represent significantly upregulated and downregulated metabolites, respectively, based on the criteria of VIP > 1, P < 0.05, and |log_2_FC| ≥ 0.26. Gray dots denote metabolites that did not meet these thresholds.

A PLS-DA model was constructed to compare the overall seminal plasma metabolic profiles between the IMI group and the CG group in both negative (ESI−) and positive (ESI+) ion modes. The PLS-DA score plots (**Figures 2A, D**) showed clear separation between the IMI and CG groups with no significant overlap of the 95% confidence ellipses. These results indicate that the two groups could be completely and effectively distinguished in both ESI− and ESI+ modes, suggesting a marked remodeling of the seminal plasma metabolic profile in patients with idiopathic infertility. To assess the risk of overfitting of the PLS-DA models, 200 random permutation tests were performed (**Figures 2B, E**). The Q² regression line intercepts in the ESI− and ESI+ modes were −0.49 and −0.46, respectively, confirming that the models were not overfitted and that the inter-group metabolic differences were robust and reliable for subsequent candidate differential metabolite screening.

Based on the combined criteria of VIP > 1, independent samples t-test *P* < 0.05, and |log_2_FC| ≥ 0.26, candidate differential metabolites were screened and visualized using volcano plots. In ESI− mode, a total of 68 candidate differential metabolites were identified, including 35 upregulated metabolites (e.g., 4-Hydroxymandelic acid, N-Palmitoyl Histidine, Neopterin) and 33 downregulated metabolites (e.g., Taurine, L-Lysine, N(6)-methyl-AMP, L-Malate) (**Figure 2C**). In ESI+ mode, a total of 152 candidate differential metabolites were identified, including 119 upregulated metabolites (e.g., PA(20:5(5Z,8Z,11Z,14Z,16E)-OH(18)/14:0), N-cis-11,14-eicosadienoyl ethanolamine, PA(20:4(7E,9E,11Z,13E)-3OH(5S,6R,15S)/18:1(9Z))) and 33 downregulated metabolites (e.g., Leu-Gly-Pro, Lys Leu Ser, 4-Oxopentanoylcarnitine) (**Figure 2F**). Following multiple-testing correction, the signal magnitudes for most metabolite-level associations were attenuated. Therefore, these findings should be interpreted with caution. The complete lists of candidate differential metabolites detected in ESI− and ESI+ modes, along with their raw *P-value*s and *P.adj*, are provided in **Supplementary Table 3.1** and **3.2**, respectively.

#### 3.2.3 KEGG enrichment and GSEA analyses

**Figure 3:**
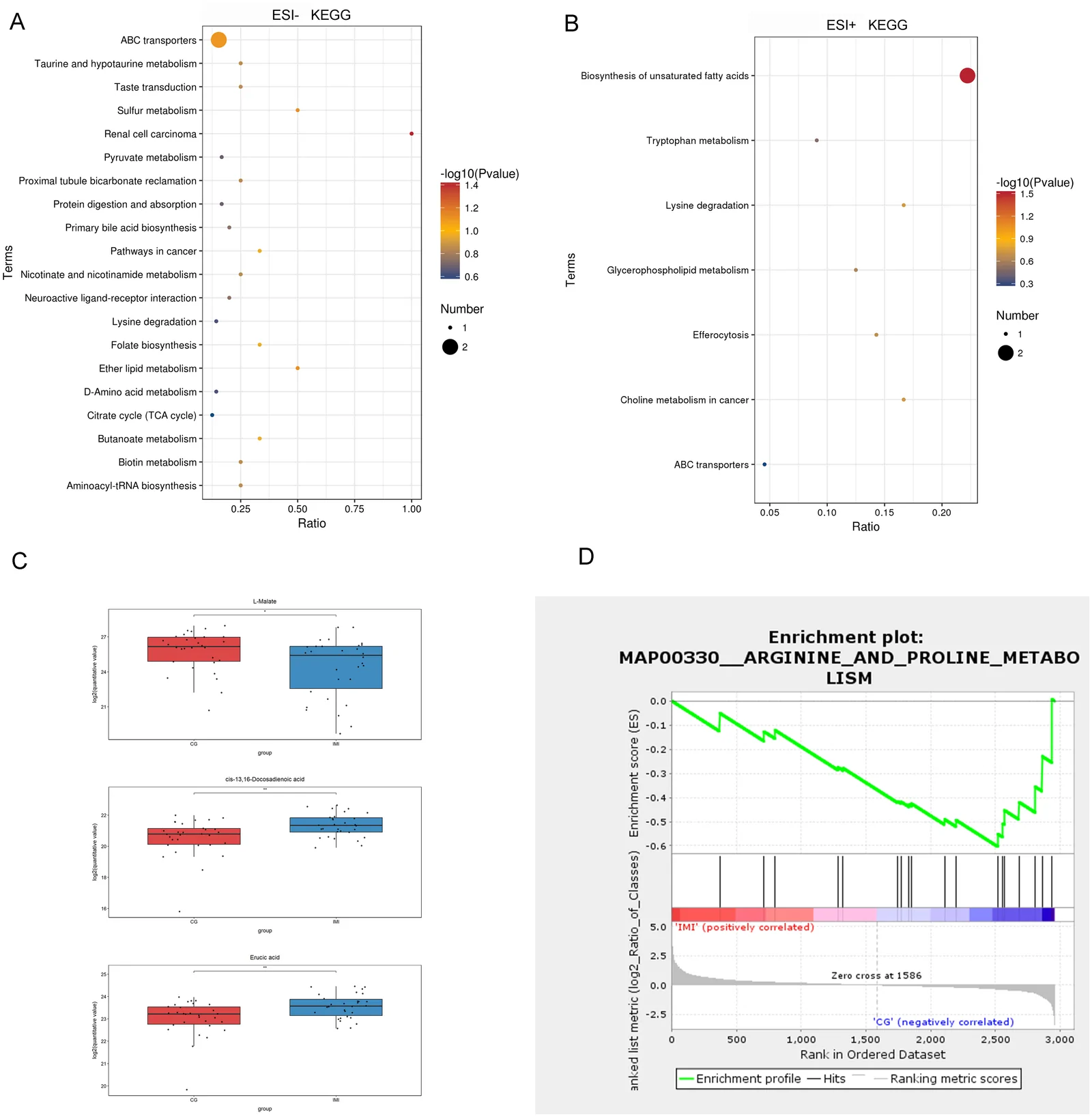
KEGG enrichment analysis and GSEA of candidate differential metabolites in seminal plasma. **(A, B)** Bubble plots of KEGG pathway enrichment based on candidate differential metabolites in ESI^-^ and ESI^+^ modes (*P* < 0.05), respectively. **(C)** Box plots showing the relative abundance of representative candidate differential metabolites, including L-malate, erucic acid and cis-13,16-docosadienoic acid. **(D)** GSEA enrichment plot of the arginine and proline metabolism pathway (MAP00330) in ESI^+^ mode. The negative enrichment score indicates that this pathway was globally suppressed in the IMI group (*P* < 0.05).

KEGG pathway enrichment analysis was performed using differentially abundant metabolites identified under ESI^-^ and ESI^+^ modes, respectively (threshold *P* < 0.05), and the results were visualized as bubble plots. Under ESI^-^ mode, the significantly enriched pathway was renal cell carcinoma (**Figure 3A**), with L-malate identified as the core differential metabolite within this pathway. Box-plot analysis showed that L-malate levels were significantly downregulated in the IMI group compared with the CG control group (**Figure 3C**). Under ESI^+^ mode, the significantly enriched pathway was biosynthesis of unsaturated fatty acids (**Figure 3B**), with erucic acid and cis-13,16-docosadienoic acid as the characteristic differential candidate metabolites; both unsaturated fatty acids were markedly upregulated in the IMI group (**Figure 3C**). Detailed results of the KEGG enrichment analysis are provided in **Supplementary Table 4.1, 4.2**. To comprehensively evaluate global pathway-level metabolic perturbations between the two groups, we further performed GSEA using the entire set of annotated metabolites. No statistically significant enrichment was observed under ESI^-^ mode. Under ESI^+^ mode, the arginine and proline metabolism pathway (MAP00330) exhibited a consistently negative enrichment profile, indicating that this pathway was globally suppressed in the IMI group (**Figure 3D**). Detailed results of the GSEA analysis are provided in **Supplementary Table 5.1, 5.2**.

#### 3.2.4 ROC curve analysis of candidate differential metabolites

**Figure 4:**
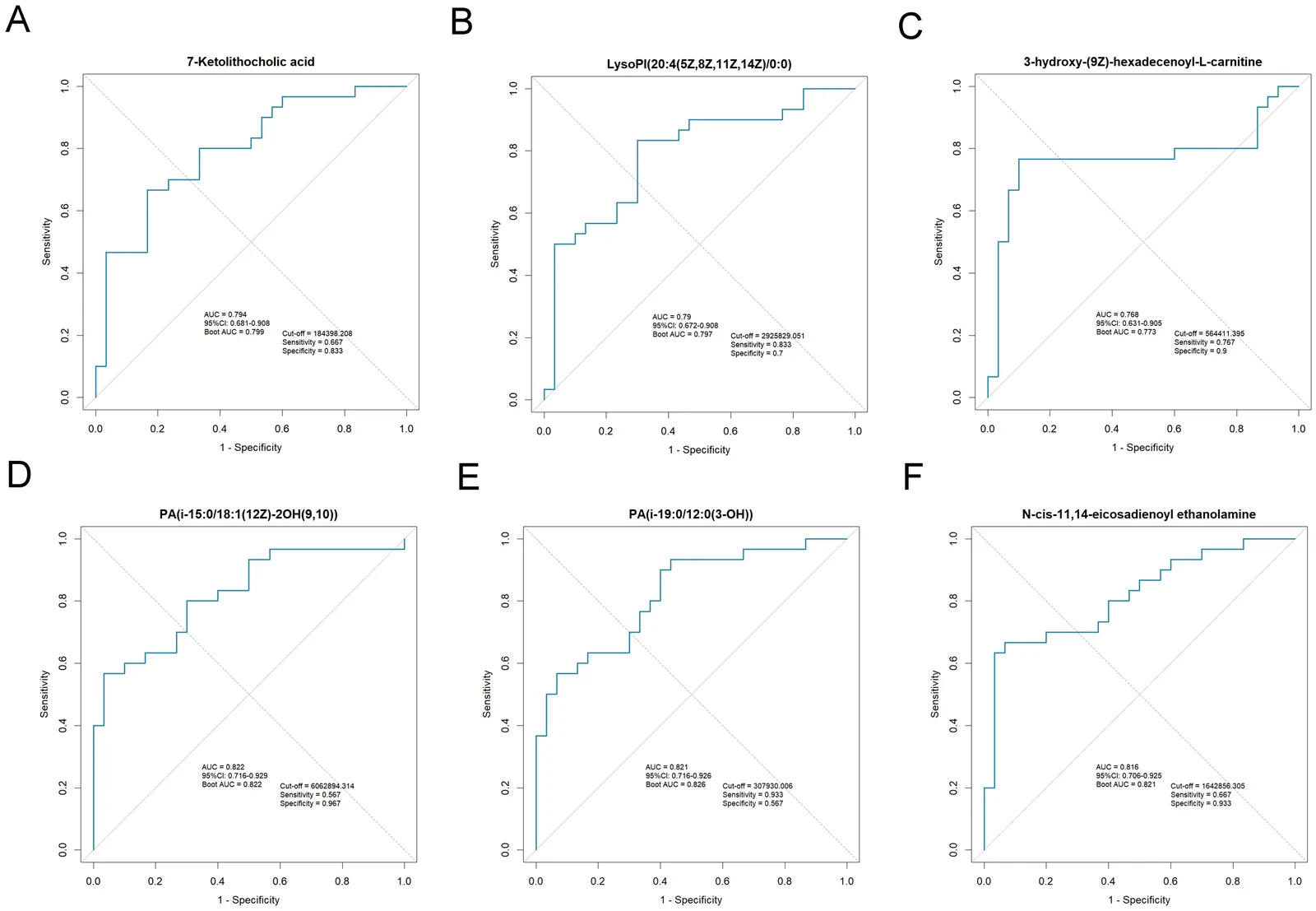
ROC curves of representative candidate differential metabolites for distinguishing IMI from CG. **(A–C)** ROC curves of the top three candidate differential metabolites with highest AUC in ESI^-^ mode:7-Ketolithocholic acid (AUC = 0.798, 95% CI: 0.681–0.908), and LysoPI(20:4(5Z,8Z,11Z,14Z)/0:0) (AUC = 0.790, 95% CI: 0.672~0.908), 3-hydroxy-(9Z)-hexadecenoyl-L-carnitine (AUC = 0.768, 95% CI: 0.631~0.905). **(D–F)** ROC curves of the top three candidate differential metabolites with highest AUC in ESI^+^ mode: PA[i-15:0/18:1(12Z)-2OH(9,10)] (AUC = 0.822, 95% CI: 0.716~0.929), PA[i-19:0/12:0(3-OH)] (AUC = 0.819, 95% CI: 0.716~0.926), and N-cis-11,14-eicosadienoyl ethanolamine (AUC = 0.816, 95% CI: 0.706~0.925).

Representative core candidate differential metabolites from the two groups were selected to generate ROC curves for evaluating their discriminatory performance (**Figure 4**). Metabolites bearing unusual natural-product-like annotations without evidence of human endogenous biosynthesis were excluded prior to analysis. In ESI^-^ mode, the top three metabolites with the highest area under the curve (AUC) were 7-Ketolithocholic acid, 3-hydroxy-(9Z)-hexadecenoyl-L-carnitine and LysoPI(20:4(5Z,8Z,11Z,14Z)/0:0), with AUC values ranging from 0.768 to 0.794. In ESI^+^ mode, the top three metabolites with the highest AUC values were PA(i-15:0/18:1(12Z)-2OH(9,10)), PA(i-19:0/12:0(3-OH)), and N-cis-11,14-eicosadienoyl ethanolamine, with AUC values ranging from 0.816 to 0.822. ROC results for metabolites under ESI^-^ and ESI^+^ modes are summarized in **Supplementary Tables 6.1** and **6.2**, respectively. All representative candidate differential metabolites yielded AUC values greater than 0.7, indicating moderate exploratory discriminatory power to differentiate men with idiopathic infertility from control subjects within this single cohort. The differentially expressed seminal metabolites identified in the present study provide preliminary molecular evidence for the exploratory development of non-invasive auxiliary biomarkers for male infertility.

### 3.3 Transcriptomic analysis of sEV-derived miRNAs

#### 3.3.1 Characterization results of sp-sEVs

**Figure 5:**
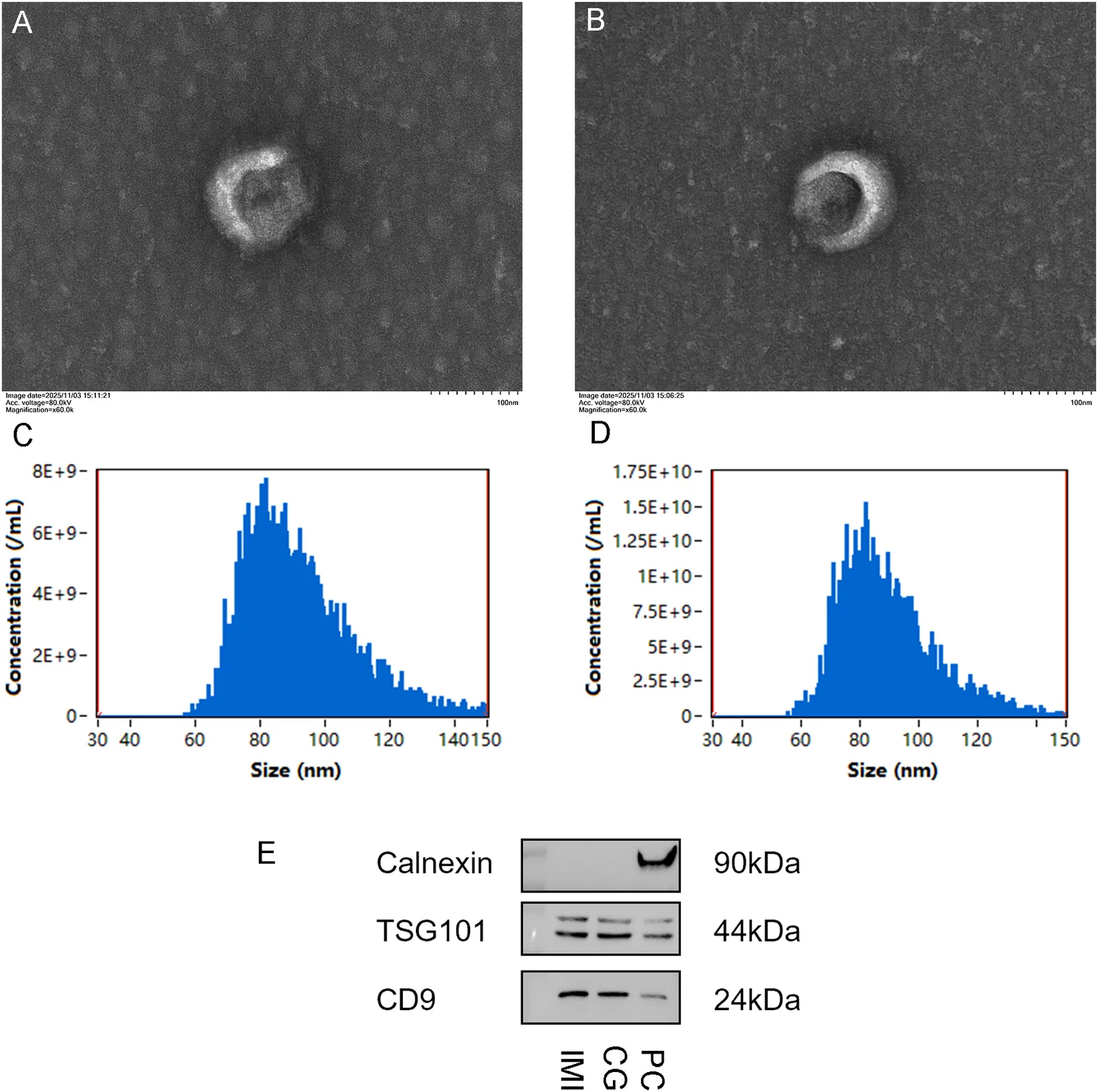
Characterization of seminal plasma small extracellular vesicles (sp-sEVs) isolated from IMI and CG. **(A, B)** Representative transmission electron microscopy (TEM) images of sp-sEVs isolated from the CG and IMI groups, showing typical cup-shaped morphology. **(C, D)** Nanoparticle size distribution and concentration of sp-sEVs isolated from the CG and IMI groups. **(E)** Western blot analysis of marker proteins TSG101 and CD9, and the negative control Calnexin.

For sp-sEVs identification, one sample from each group was selected for morphological, nanoparticle tracking and western blotting characterization. Transmission electron microscopy (TEM) images revealed that the isolated EVs exhibited typical cup-shaped or disc-shaped morphology (**Figures 5A, B**). The EVs identification results showed that the mean diameter of sp-sEVs in the IMI group was approximately 89 nm with a concentration of 7.88 × 10^11^ particles/mL, while those in the CG group had a mean diameter of approximately 91.5 nm with a concentration of 4.59 × 10^11^ particles/mL. The particle size distribution and concentration profiles are shown in **Figures 5C, D**.

Western blotting was performed to characterize the isolated EVs. The marker proteins TSG101 and CD9 were clearly detected in both the CG and IMI groups, as well as in the positive control (PC). In contrast, the endoplasmic reticulum protein Calnexin was expressed only in the positive control samples and was absent in both the CG and IMI groups, indicating that the isolated EV samples were free from contamination by cell debris or intracellular organelles. Collectively, the vesicles isolated from both groups exhibited typical protein profiles, confirming their identity and purity for subsequent functional studies (**Figure 5E**).

#### 3.3.2 Summary of small RNA library sequencing

The raw read counts of the 10 semen samples in the CG group ranged from 10,090,611 to 12,573,853, with effective reads ranging from 9,821,189 to 11,965,320 after quality control, and Q30 values ranging from 98.11% to 98.69%. In the IMI group, the raw read counts ranged from 10,158,548 to 11,690,783, with effective reads ranging from 9,652,510 to 11,232,125 after quality control, and Q30 values ranging from 98.58% to 98.78%. All samples had Q30 values exceeding 97%, indicating high sequencing data quality and reliability for subsequent analyses (**Table 2**).

**Table 2:** Summary of miRNA sequencing data quality from sp-sEVs in the CG and IMI groups.

Group	Raw reads	Clean reads	Q30 (%)	Reads retention rate (%)	Total sRNA reads	Unique sRNA reads
CG	10,090,611~12,573,853	9,821,189~11,965,320	98.11~98.69	92.01 ~ 95.12	8,852,669~11,602,244	229,006~368,282
IMI	10,158,548~11,690,783	9,652,510~11,232,125	98.58~98.78	93.15 ~ 96.08	8,228,122~10,392,400	193,292~458,387

**Figure 6:**
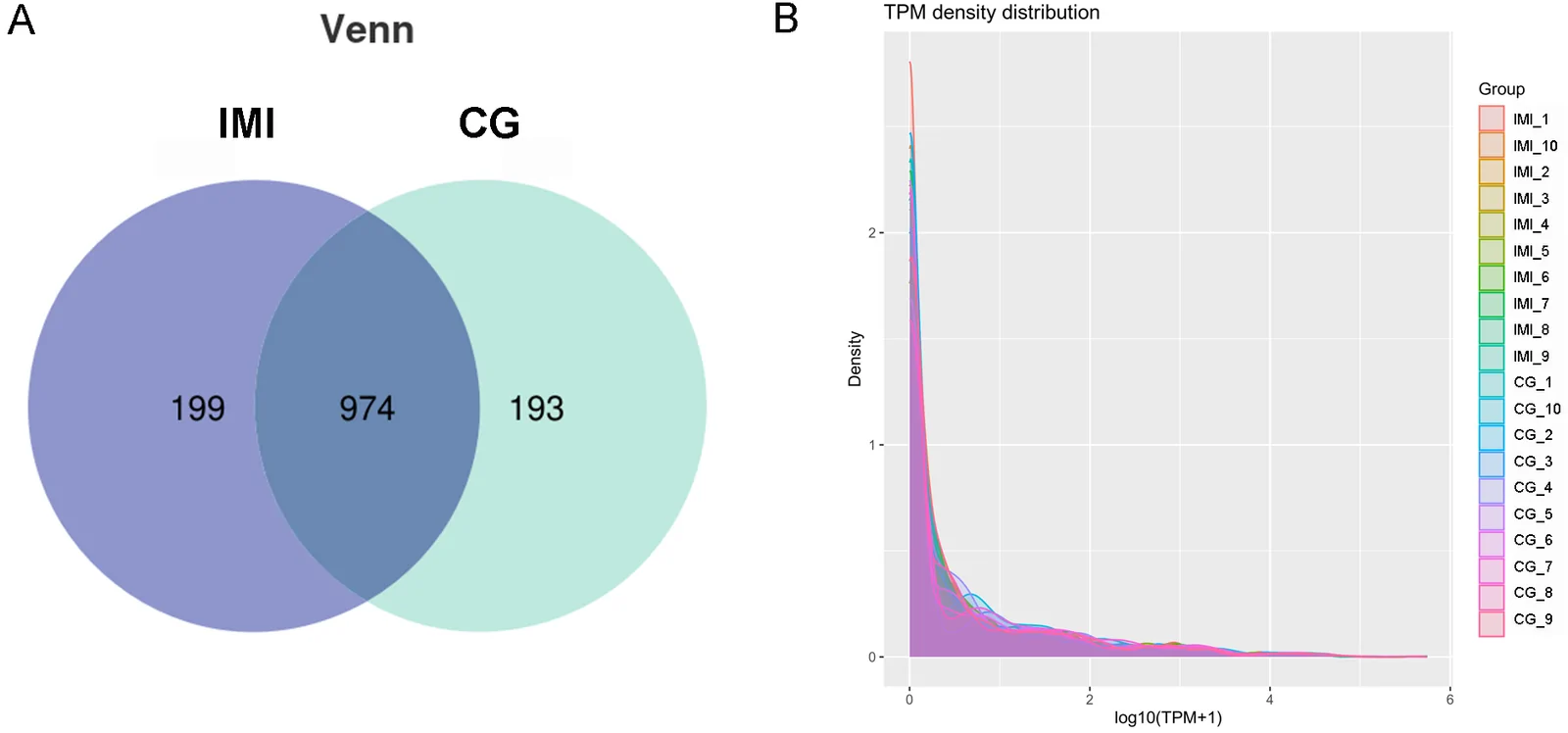
Overview of miRNA expression profiles in sp-sEVs from CG and IMI groups. **(A)** Venn diagram showing the number of co-expressed and group-specific miRNAs between the two groups. **(B)** TPM density distribution curves of all samples, showing consistent expression patterns across groups, indicating good data uniformity and reproducibility.

A total of 1,271 mature miRNAs and 1,113 hairpin precursors were identified, with 95 novel mature miRNAs and 98 novel hairpin precursors predicted. In total, 1,366 miRNAs were identified, of which 974 were co-expressed in both groups, 199 were specifically expressed in the IMI group, and 193 were specifically expressed in the CG group (**Figure 6A**). The TPM density distribution curves showed consistent expression patterns across all samples, indicating good data uniformity and stable overall expression profiles among samples, which were suitable for subsequent differential expression analysis (**Figure 6B**).

#### 3.3.3 Differentially expressed miRNAs between groups

**Figure 7:**
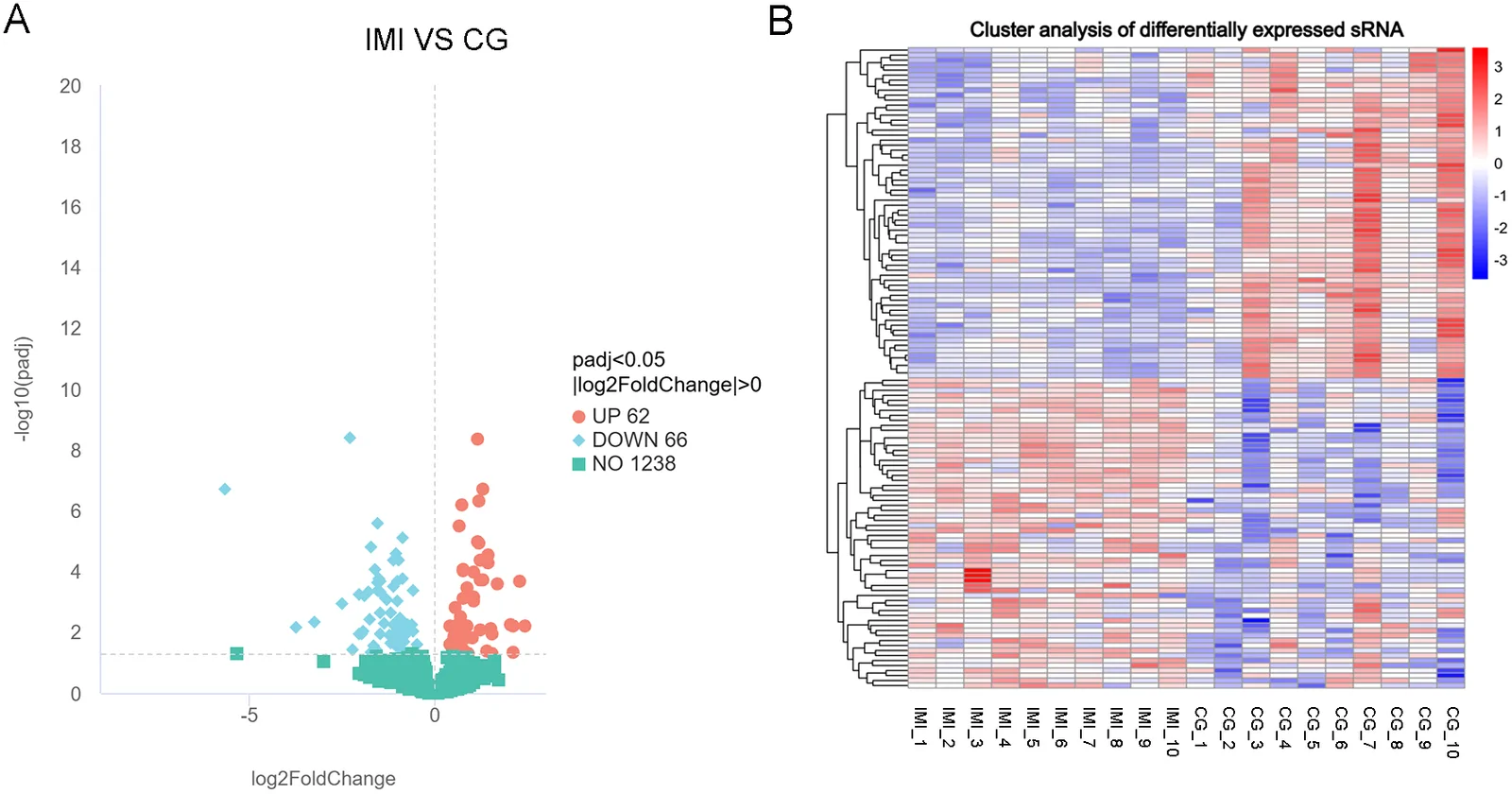
Differentially expressed miRNAs between IMI and CG groups. **(A)** Volcano plot of differentially expressed miRNAs. Red and blue dots represent significantly upregulated and downregulated miRNAs in the IMI group, respectively (|log_2_FC| > 0, *P* < 0.05). Gray dots indicate miRNAs that did not meet the significance thresholds. **(B)** Hierarchical clustering heatmap of differentially expressed miRNAs showing clear separation between IMI and CG samples. Red and blue represent high and low expression levels, respectively.

Differential expression analysis identified 128 miRNAs that were significantly altered between the IMI and CG groups (**Figure 7A**), of which 62 were upregulated (e.g., hsa-miR-487b-3p, hsa-miR-200c-5p, hsa-miR-4454) and 66 were downregulated (e.g., hsa-miR-302a-5p, hsa-miR-302b-3p, hsa-miR-328-5p) in the IMI group. Hierarchical clustering based on these differentially expressed miRNAs clearly distinguished the two groups (**Figure 7B**), indicating substantial divergence in miRNA expression profiles between the two groups. The full list of differentially expressed miRNAs, together with raw *P-values* and adjusted *P.adj*, are provided in **Supplementary Table 7**.

#### 3.3.4 Target gene prediction and enrichment analysis

**Figure 8:**
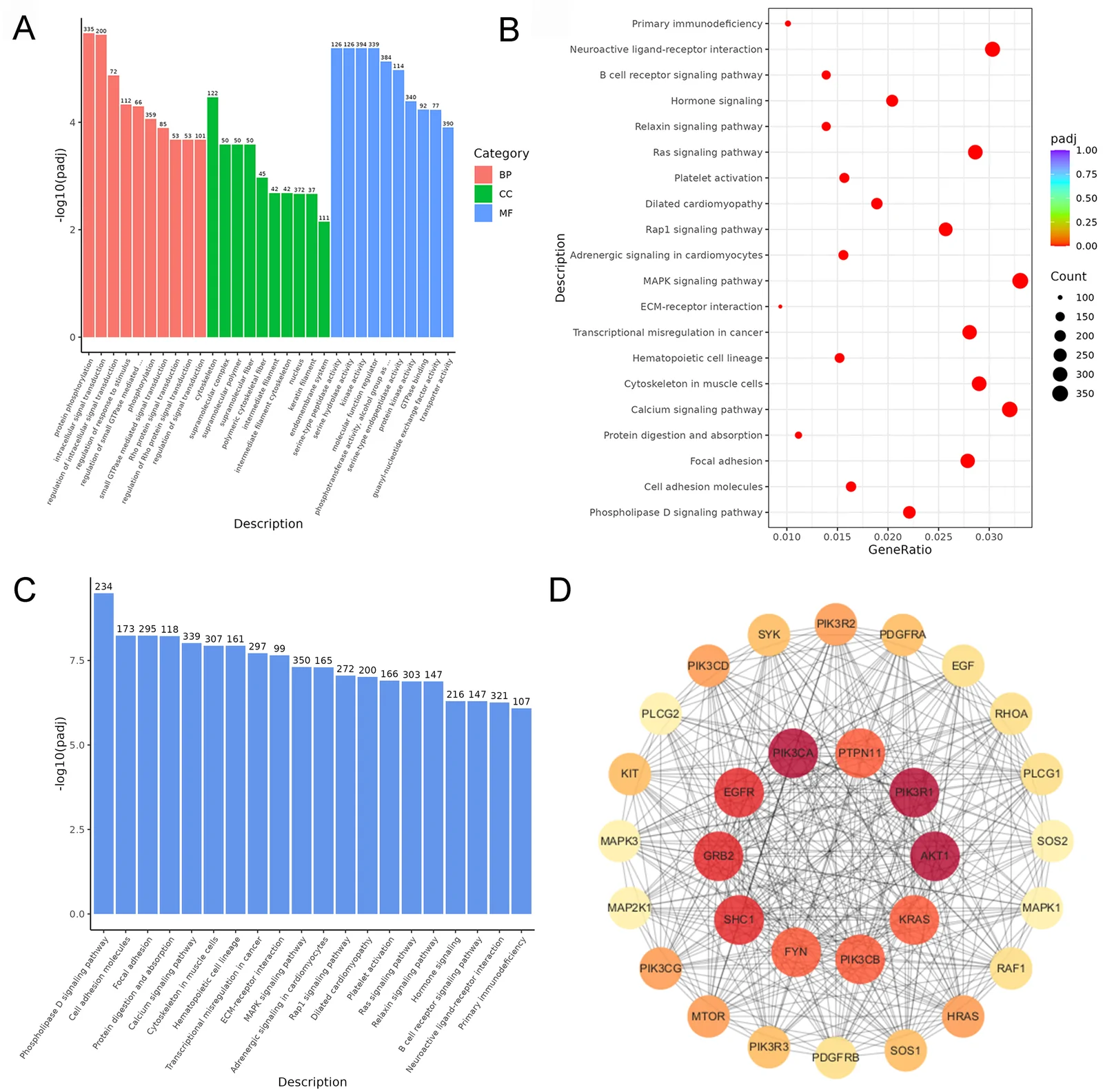
GO and KEGG enrichment analyses of miRNA target genes and PPI network of PLD signaling pathway-related targets. **(A)** GO enrichment analysis of target genes, showing the top enriched terms in biological process (BP), cellular component (CC), and molecular function (MF) categories. **(B, C)** KEGG pathway enrichment analysis of target genes, with the phospholipase D (PLD) signaling pathway, MAPK signaling pathway, and calcium signaling pathway among the most significantly enriched terms, and the PLD signaling pathway showing the highest enrichment significance. **(D)** Protein-protein interaction (PPI) network of PLD signaling pathway-related target genes constructed using Cytoscape. The top 30 hub genes were screened via the MCC algorithm, with *PIK3CA*, *PIK3R1*, and *AKT1* identified as the core hub genes.

Target genes of differentially expressed miRNAs were predicted using the miRDB database, and subsequent GO and KEGG enrichment analyses were performed on the target gene set. GO functional enrichment analysis revealed that the biological process (BP) category was predominantly enriched in protein phosphorylation and intracellular signal transduction; the cellular component (CC) category was enriched in cytoskeleton and supramolecular complex; and the molecular function (MF) category was enriched in serine-type peptidase activity and kinase activity (**Figure 8A**). KEGG pathway enrichment analysis identified the phospholipase D (PLD) signaling pathway, MAPK signaling pathway, and calcium signaling pathway among the top enriched terms (**Figure 8B**). The bar plot (**Figure 8C**) showed the −log10(*P.adj*) values, with the PLD signaling pathway exhibiting the lowest *P.adj* and thus the highest enrichment significance. Detailed results of GO enrichment and KEGG pathway enrichment analyses are provided in **Supplementary Tables 8.1, 8.2**, respectively. To further dissect the interactions among target genes within the PLD signaling pathway, PLD pathway-related target genes were imported into the STRING database to obtain PPI information. The top 30 hub genes were screened using the MCC algorithm, and the PPI network was constructed using Cytoscape software (**Figure 8D**). The network revealed *PIK3CA*, *PIK3R1*, and *AKT1* as the core hub genes.

### 3.4 Correlation analysis between sp-sEV miRNAs and candidate differential metabolites

**Figure 9:**
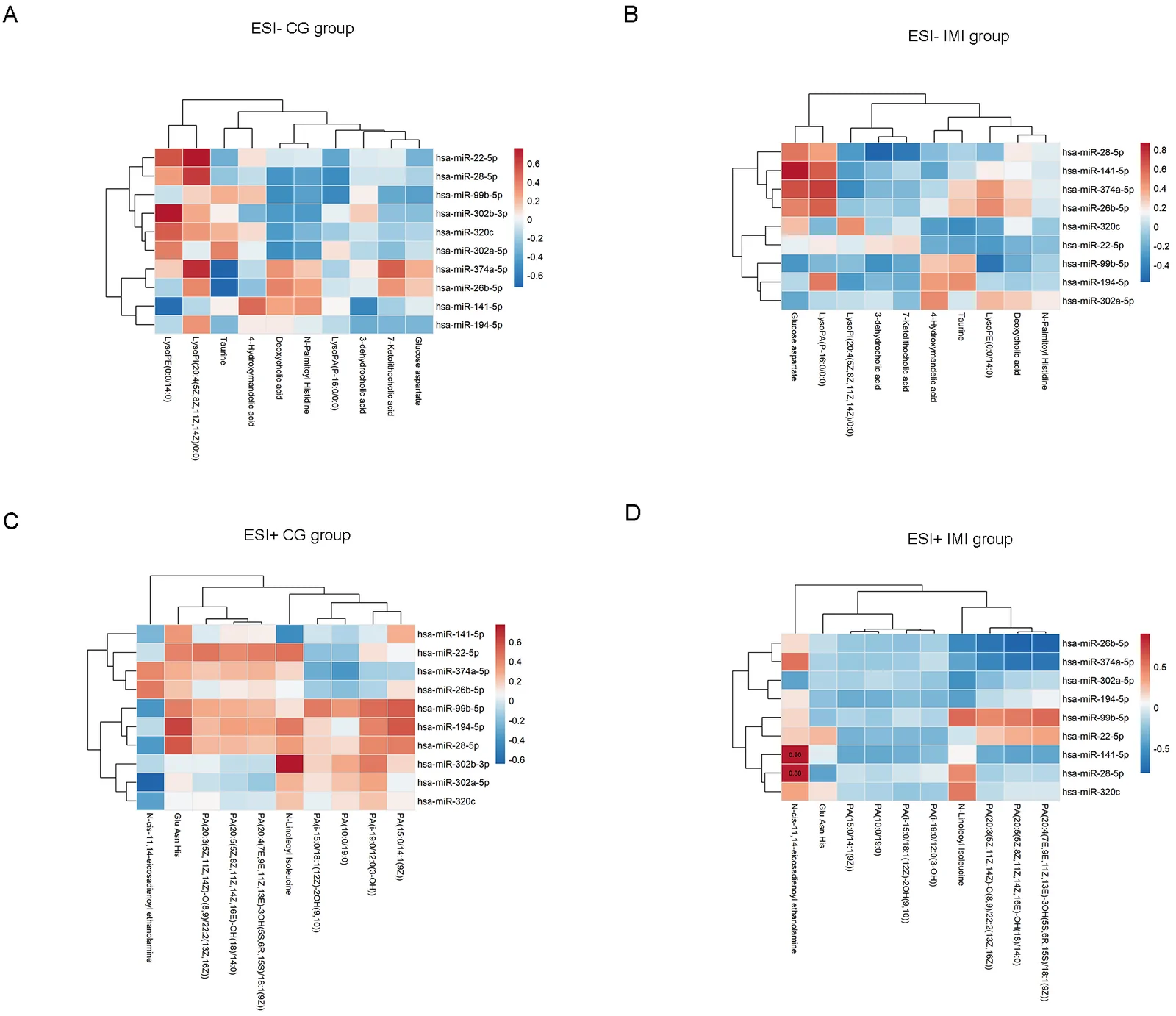
Heatmaps of Pearson correlation coefficients between key differentially expressed sp-sEV miRNAs and seminal plasma candidate differential metabolites. **(A)** Correlation heatmap for metabolites detected under ESI^-^ mode in CG group; **(B)** Correlation heatmap for metabolites detected under ESI^-^ mode in IMI group; **(C)** Correlation heatmap for metabolites detected under ESI^+^ mode in CG group; **(D)** Correlation heatmap for metabolites detected under ESI^+^ mode in IMI group. Rows represent candidate miRNAs isolated from sp-sEVs, columns represent candidate differential metabolites. Color gradient indicates the value of Pearson correlation coefficient: red represents positive correlation, blue represents negative correlation. Hierarchical clustering was performed on both rows and columns based on correlation distance.

Pairwise correlations were screened using unadjusted (*P* < 0.05, |r| > 0.6) as preliminary thresholds, and Benjamini–Hochberg FDR correction was performed to eliminate false positives derived from multiple comparisons. Correlation heatmaps were plotted to visualize correlation patterns under ESI^-^ and ESI^+^ modes (**Figure 9**). In the CG group, several miRNA-metabolite pairs met the preliminary screening criteria: hsa-miR-302b-3p was positively correlated with N-Linoleoyl Isoleucine (*r* = 0.778, *P* = 0.008), and hsa-miR-302a-5p showed negative correlation with N-cis-11,14-eicosadienoyl ethanolamine (*r* = -0.643, *P* = 0.045). However, neither pair remained statistically significant after FDR adjustment. For the IMI group, hsa-miR-26b-5p and hsa-miR-374a-5p exhibited prominent negative correlations with multiple hydroxylated polyunsaturated phosphatidic acid derivatives, including PA (20:4(7E,9E,11Z,13E)-3OH(5S,6R,15S)/18:1(9Z)). Following FDR correction, only two correlated pairs retained statistical significance, both associated with N-cis-11,14-eicosadienoyl ethanolamine. hsa-miR-141-5p showed a strong positive correlation with this metabolite (*r* = 0.903, *P* = 0.00035, *FDR* = 0.0315), and hsa-miR-28-5p also displayed a significant positive correlation (*r* = 0.877, *P* = 0.00086, *FDR* = 0.0388). Detailed correlation results, stratified by ion mode and group, are available in **Supplementary Table 9.1** (ESI-, CG), 9.2 (ESI-, IMI), 9.3 (ESI+, CG), and 9.4 (ESI+, IMI).

## 4 Discussion

IMI refers to a condition of impaired semen quality in which no specific etiological factors have been identified. Due to its complex etiology and substantial inter-individual variability, the pathogenesis of IMI remains incompletely understood. Accumulating evidence has demonstrated significant differences in seminal plasma metabolites between idiopathic infertile men and normozoospermic controls, involving multiple pathways such as lipid metabolism and amino acid biosynthesis. However, the underlying mechanisms driving these metabolic abnormalities in IMI patients have rarely been explored. In this study, we innovatively adopted a nested matched-subgroup strategy within the same cohort, integrating untargeted metabolomics and sp-sEV miRNA sequencing to systematically characterize the molecular disturbances in the seminal plasma microenvironment of IMI patients.

### 4.1 Metabolomic profiling of seminal plasma in IMI patients

Although several studies have conducted metabolomic analyses of seminal plasma in idiopathic infertile males, the reported findings have been inconsistent, which may be attributable to differences in detection methods and study populations. In the present study, we performed metabolomic profiling of seminal plasma from 30 IMI patients and 30 healthy controls in both positive and negative ion modes. A total of 2,959 metabolites were identified in positive ion mode and 2,123 in negative ion mode. In both modes, the most abundant metabolite categories were lipids and lipid-like molecules, organic acids and derivatives, and organoheterocyclic compounds. Differential metabolite analysis revealed downregulation of taurine, L-malate, and L-lysine, and upregulation of erucic acid and multiple phosphatidic acid (PA) species. Collectively, these metabolic features show partial concordance with a phenotype of enhanced lipid peroxidation, and reduced antioxidant capacity, yet direct functional evidence remains absent from this study.

Taurine, one of the most abundant amino acids in the male reproductive system, exerts multiple functions including antioxidant, anti-apoptotic, osmoregulatory, and calcium-modulating effects. Taurine supplementation has been shown to significantly improve sperm motility, viability, and plasma membrane integrity, and to prolong sperm survival *in vitro* (32–34). L-Malate, a key intermediate of the tricarboxylic acid (TCA) cycle, is directly involved in ATP production and can activate pyruvate dehydrogenase (PDH), a key mitochondrial enzyme, thereby enhancing metabolic efficiency (35). L-Lysine is an essential amino acid required for spermatogenesis. Studies have demonstrated that post-translational modifications (PTMs) occurring on L-lysine residues are critical for the regulation of sperm function (36), and zinc-lysine supplementation has been shown to significantly improve sperm motility (37). Erucic acid, a 22-carbon long-chain monounsaturated fatty acid predominantly found in plant oils such as rapeseed oil, has been reported by Jiaming S et al. (38) to impair male fertility by inhibiting retinoic acid synthesis in Sertoli cells. In addition, our study found upregulation of multiple PA species in the seminal plasma of the IMI group. Correnti S et al. (39) based on UHPLC-MS lipidomic analysis of seminal plasma, reported significant elevation of certain PA species in infertile men. PA can contribute to reactive oxygen species (ROS) production by activating NADPH oxidase (40), and has also been implicated in the coordination of mitochondrial dynamics (41). The metabolic signature detected in IMI seminal plasma is compatible with an oxidative-stress-associated metabolic phenotype, although markers of oxidative stress (OS) were not directly quantified in our study. Moderate ROS concentrations support physiological processes including sperm maturation, capacitation and the acrosome reaction; conversely, excessive ROS production exceeding endogenous antioxidant buffering capacity may contribute to mitochondrial dysfunction, lipid peroxidation and sperm DNA fragmentation (42).

KEGG enrichment analysis of candidate differential metabolites revealed significant enrichment of the renal cell carcinoma pathway, with L-malate as the key metabolite within this pathway. The biosynthesis of unsaturated fatty acids pathway was also significantly enriched, with erucic acid and cis-13,16-docosadienoic acid as the characteristic metabolites. In addition, metabolites were also enriched in the ABC transporters pathway, although this did not reach statistical significance; nonetheless, this pathway may directly affect sperm maturation, capacitation, and ultimately fertilizing capacity through precise regulation of lipid metabolism (43). To comprehensively evaluate global pathway-level perturbations, we performed GSEA on all annotated metabolites. The results showed that the arginine and proline metabolism pathway (MAP00330) consistently exhibited a negative enrichment profile, indicating global suppression of this pathway in the IMI group. Previous studies have identified this pathway as a differential metabolic pathway associated with semen preservation capacity (44). Taken together, these pathway-level perturbations may offer a mechanistic framework to interpret the seminal-plasma features observed in IMI patients. The relatively small number of significantly enriched pathways may reflect substantial inter-individual heterogeneity among IMI patients, as well as the modest sample size of the present study. Even so, characteristic discriminatory metabolites yielded AUC values above 0.70 in both ion modes, hinting at their prospective utility as non-invasive auxiliary biomarkers for IMI and providing exploratory metabolomic evidence for future biomarker development.

### 4.2 miRNA profiling of sp-sEVs in idiopathic male infertility

In this study, a total of 1,366 miRNAs were identified through sp-sEV miRNA sequencing, of which 128 were differentially expressed, including 62 upregulated and 66 downregulated in the IMI group. Several miRNAs in the differential expression list have been previously implicated in male reproduction (45), For instance, Ma J et al. found that miR-574 was highly expressed in spermatozoa of aged mice and demonstrated that miR-574 suppresses mitochondrial function and reduces ATP production in GC2 cells (46); Cito G et al. reported that plasma miR-20a-5p was significantly elevated in patients with non-obstructive azoospermia (NOA) (47); Yu Y et al. found that fluoride exposure could enhance oxidative stress, induce inflammatory responses, increase lipid synthesis, and reduce lipid breakdown and transport in the caudal epididymis, with miR-141-5p identified as a key regulatory factor in this process (48). GO enrichment analysis of target genes predicted from differentially expressed miRNAs revealed that the top enriched BP terms included protein phosphorylation and intracellular signal transduction; the top CC terms included cytoskeleton and supramolecular complex; and the top MF terms included serine-type peptidase activity and kinase activity. KEGG dot plot analysis showed that the MAPK signaling pathway and calcium signaling pathway accounted for the highest proportions among target genes, while the bar plot indicated that the phospholipase D (PLD) signaling pathway exhibited the highest enrichment significance. Therefore, we constructed a PPI network based on target genes within the PLD signaling pathway, which revealed *PIK3CA* and *AKT1* as core hub genes. Both *PIK3CA* and *AKT1* have been demonstrated to participate in biological processes such as sperm capacitation (49). PLD plays a critical role in sperm function as a class of signaling enzymes that hydrolyze phosphatidylcholine (PC) to generate the important lipid second messenger PA (50). Notably, this PLD-pathway-centered miRNA-target signature is concordant with our metabolomic finding of elevated PA species in IMI seminal plasma. Nevertheless, PA can be produced via multiple enzymatic routes apart from PLD-mediated catalysis. On the basis of the above correlative findings, we hypothesize that sp-sEV-derived miRNAs may contribute to lipid-metabolism remodeling partially through the PLD-PA regulatory axis, although functional validation is required to substantiate this inference.

### 4.3 Correlation analysis between sp-sEV miRNAs and candidate differential metabolites

Correlation analyses were performed on nested matched-subgroup samples, and the pairwise molecular associations between miRNAs and metabolites were visualized using a correlation heatmap. Several nominal associations were observed prior to multiple testing adjustment: for instance, upregulated hsa-miR-141-5p and hsa-miR-194-5p displayed negative trends with 7-ketolithocholic acid and positive trends with LysoPA(P-16:0/0:0), while downregulated hsa-miR-302a-5p and hsa-miR-99b-5p tended to correlate negatively with N-cis-11,14-eicosadienoyl ethanolamine. Nevertheless, most of these nominal correlations lost statistical significance after Benjamini-Hochberg FDR correction. Only two pairs retained robust statistical significance following correction, both linked to N-cis-11,14-eicosadienoyl ethanolamine: hsa-miR-141-5p and hsa-miR-28-5p showed positive correlation. N-cis-11,14-eicosadienoyl ethanolamine is a fatty amide obtained by the formal condensation of (11Z,14Z)-eicosadienoic acid with ethanolamine. It is a fatty amide and functionally related to an (11Z,14Z)-icosadienoic acid, there is limited research on this metabolite, currently (51).

In summary, through a nested multi-omics integration strategy, this study systematically delineated the coordinated alterations of metabolites and sp-sEV miRNAs in the seminal plasma microenvironment of patients with IMI. Metabolomics revealed a metabolic remodeling characterized by perturbations in the biosynthesis of unsaturated fatty acids and the arginine and proline metabolism pathway in IMI seminal plasma, with significant downregulation of protective metabolites such as taurine, L-malate, and L-lysine, and marked accumulation of erucic acid and multiple PA species. These metabolic signatures may suggest a perturbed microenvironment in IMI seminal plasma, potentially featuring elevated oxidative stress burden, compromised mitochondrial energy metabolism, and accumulated lipotoxic lipid species as inferred from the observed metabolite shifts. It is worth reiterating that most nominal correlative signals became non-significant after FDR correction, so the relevant results should be interpreted prudently. Transcriptomics further revealed that target genes of differentially expressed miRNAs in IMI sp-sEVs were significantly enriched in the PLD signaling pathway, with *PIK3CA*, *PIK3R1*, and *AKT1* constituting the core hubs of the protein-protein interaction network. As PLD is the principal enzymatic source of PA generation, these correlations raise the hypothesis that sp-sEV miRNAs may influence PA metabolism via the PLD pathway, but this model requires functional validation, including PLD activity assays, target gene expression analysis, and miRNA-target binding experiments. Among the candidate metabolites, PA(i-15:0/18:1(12Z)-2OH(9,10)) exhibited promising exploratory discriminatory performance (AUC > 0.8) in this single cohort, suggesting its potential value as a candidate molecule for further evaluation in non-invasive IMI biomarker studies. Nevertheless, this study provides only preliminary exploratory findings; external multicenter cohorts are indispensable for rigorous validation prior to any clinical translation.

### 4.4 Study limitations

Several limitations of this study should be acknowledged. First, the sample size was relatively modest (metabolomics cohort, n = 60; transcriptomics cohort, n = 20), and further validation in larger cohorts is warranted. Second, the miRNA-metabolite correlations were based on Pearson analysis, and causal regulatory relationships require confirmation through cellular and animal experiments. Third, proteomic data of sp-sEVs were not included, leaving the upstream and downstream signaling nodes of the PLD pathway incompletely covered. Fourth, our metabolomic analysis primarily focused on endogenous metabolites; exogenous metabolites, such as drug residues and environmental pollutants that may be present in the untargeted metabolomic data, were not systematically identified or discussed, which may lead to an incomplete assessment of dietary and environmental exposure-related pathogenic factors in IMI. Fifth, this is an exploratory multi-omics study, a portion of correlative outcomes were not adjusted by FDR correction, and such findings must be interpreted with considerable caution. Sixth, limited by the preciousness of clinical samples, characterization of sp-sEVs was only conducted using one specimen per group, quantitative analysis of extracellular vesicle abundance could not be carried out accordingly, and future studies with more comprehensive characterization are warranted.

## Data Availability

The raw transcriptome sequencing data generated in this study have been deposited in the Human Research Archive (HRA), China National Center for Bioinformation, under accession number HRA020502. The metabolomics data have been deposited in OMIX under accession number OMIX019846.
