# Supplementary material for: Cross-omics association of small extracellular vesicle miRNAs and metabolites in seminal plasma of idiopathic male infertility
Source: Front Endocrinol (Lausanne). 2026 Sep 9;17:1935941. doi: 10.3389/fendo.2026.1935941 (PMC13597263; doi:10.3389/fendo.2026.1935941)
Supplement: Supplementary file 1 [file DataSheet1.zip › Supplementary Figures/CG_NTA.pdf]

Size & Concentration Report

15 3000×

Data File	20251105 15 3000× 21.nfa	Population	Total

SN: FNAN30E20071514
Software: V2.0
Sample Pressure: 1.0Kpa

Laser: 5/40 mW 488
SS Decay: 10%
Threshold/sub: 68.9 7.8 1 1/0 0 0 0
Min Width: 0.3 ms

Total Size Infomation

Gating Range 30 - 150 nm

All Events	5038	
Gating Events	4999	
% of all	99.23	
Median	88.2	nm
Mean	91.5	nm
Std Dev.	16.4	nm

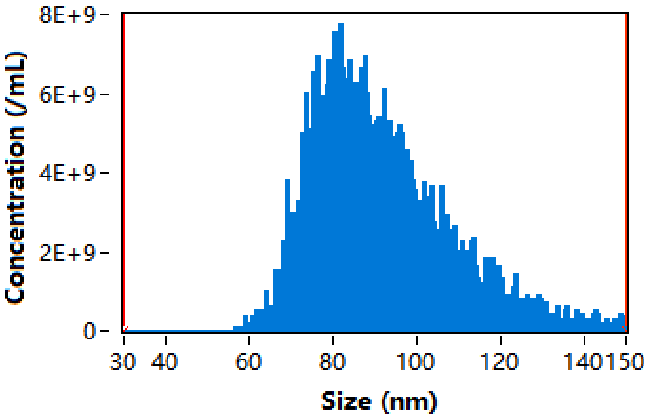

Total Concentration Infomation

	Particle Number	Dilution Factor
STD	7111	100
Blank	45	———
Sample	5083	3000

STD Con.	2.16E+10	Particles/mL
Sample Flow Rate	32.92	nL/min
Sample Con.	4.59E+11	Particles/mL

Corrected Ratio: 5038/5038 100.0%

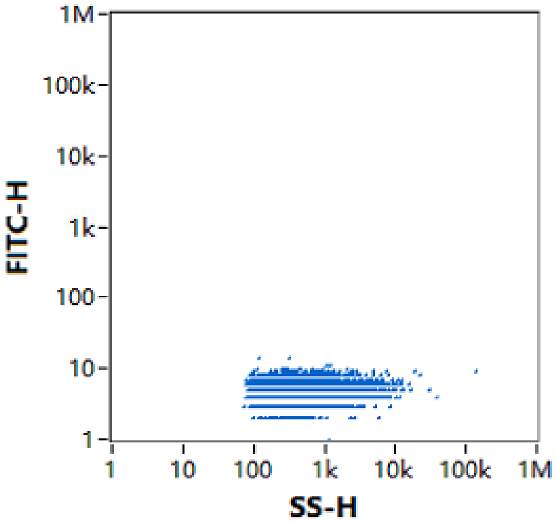

Report By :

2025/11/5 13:49
