# Supplementary material for: Cross-omics association of small extracellular vesicle miRNAs and metabolites in seminal plasma of idiopathic male infertility
Source: Front Endocrinol (Lausanne). 2026 Sep 9;17:1935941. doi: 10.3389/fendo.2026.1935941 (PMC13597263; doi:10.3389/fendo.2026.1935941)
Supplement: Supplementary file 1 [file DataSheet1.zip › Supplementary Figures/concentration standard beads.pdf]

Concentration Report

250 nm Std FL SiNPs

Data File	20251105 250 nm Std FL SiNPs 1.nfa	Population	Total

SN: FNAN30E20071514
Software: V2.0
Sample Pressure: 1.1Kpa

Laser: 20/40 mW 488
SS Decay: 0.2%
Threshold/sub: 64.4 7.9 1 1/0 0 0 0
Min Width: 0.3 ms

Total Concentration Infomation

	Particle Number	Dilution Factor
STD	7111	100
Blank	47	——
Sample	7215	100

STD Con.	2.16E+10	Particles/mL
Sample Flow Rate	32.92	nL/min
Sample Con.	2.18E+10	Particles/mL

Corrected Ratio: 7168/7168 100.0%

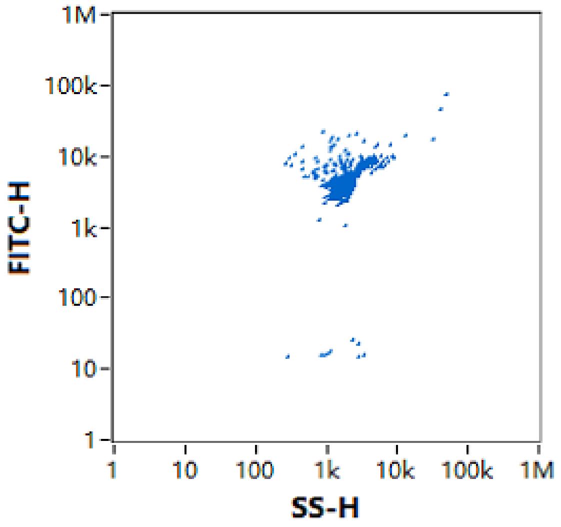

Blank

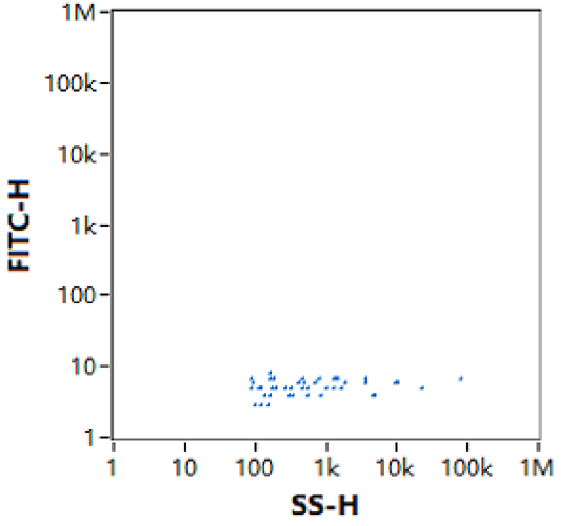

STD

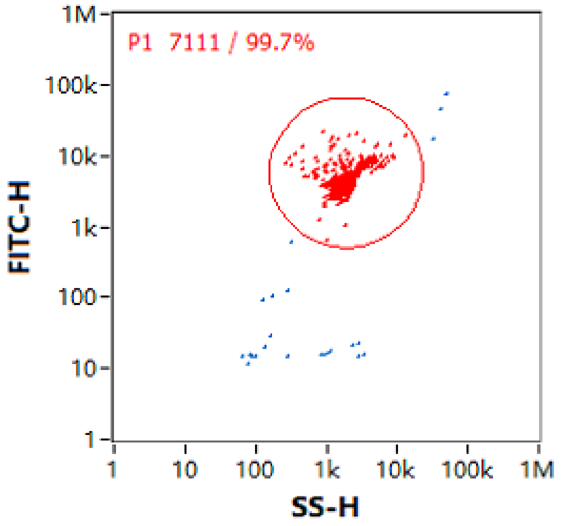

Report By :

2025/11/5 9:50
