# Supplementary material for: Cross-omics association of small extracellular vesicle miRNAs and metabolites in seminal plasma of idiopathic male infertility
Source: Front Endocrinol (Lausanne). 2026 Sep 9;17:1935941. doi: 10.3389/fendo.2026.1935941 (PMC13597263; doi:10.3389/fendo.2026.1935941)
Supplement: Supplementary file 1 [file DataSheet1.zip › Supplementary Figures/Figure S1.docx]

Calnexin

TSG101
CD9
