# Supplementary material for: Cross-omics association of small extracellular vesicle miRNAs and metabolites in seminal plasma of idiopathic male infertility
Source: Front Endocrinol (Lausanne). 2026 Sep 9;17:1935941. doi: 10.3389/fendo.2026.1935941 (PMC13597263; doi:10.3389/fendo.2026.1935941)
Supplement: Supplementary file 1 [file DataSheet1.zip › Supplementary Figures/IMI_NTA.pdf]

Size & Concentration Report

14 6000×

Data File: 20251105 14 6000× 20.nfa
Population: Total

SN: FNAN30E20071514
Software: V2.0
Sample Pressure: 1.0Kpa

Laser: 5/40 mW 488
SS Decay: 10%
Threshold/sub: 67.4 7.8 1 1/0 0 0 0
Min Width: 0.3 ms

Total Size Infomation

Gating Range 30 - 150 nm

All Events	4326	
Gating Events	4311	
% of all	99.65	
Median	86.2	nm
Mean	89.0	nm
Std Dev.	15.6	nm

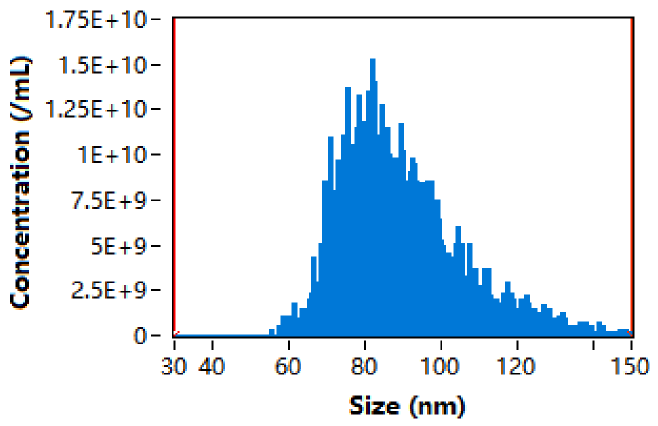

Total Concentration Infomation

	Particle Number	Dilution Factor
STD	7111	100
Blank	46	——
Sample	4372	6000

STD Con.	2.16E+10	Particles/mL
Sample Flow Rate	32.92	nL/min
Sample Con.	7.88E+11	Particles/mL

Corrected Ratio: 4326/4326 100.0%

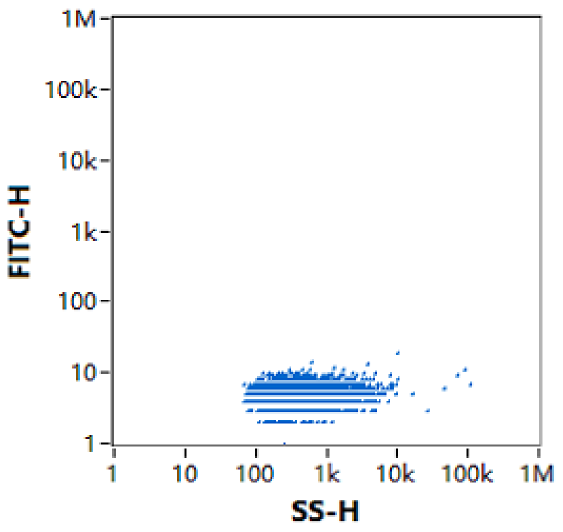

Report By :

2025/11/5 13:44
