# Supplementary material for: Cross-omics association of small extracellular vesicle miRNAs and metabolites in seminal plasma of idiopathic male infertility
Source: Front Endocrinol (Lausanne). 2026 Sep 9;17:1935941. doi: 10.3389/fendo.2026.1935941 (PMC13597263; doi:10.3389/fendo.2026.1935941)
Supplement: Supplementary file 1 [file DataSheet1.zip › Supplementary Figures/size standard beads.pdf]

Size Report

68-155 S16M-Exo

Data File: 20251105 68-155 S16M-Exo 2.nfa   Population: Total

SN: FNAN30E20071514
Software: V2.0
Sample Pressure: 1.0Kpa

Laser: 5/40 mW 488
SS Decay: 10%
Threshold/sub: 64.4 7.9 1 1/0 0 0 0
Min Width: 0.3 ms

Total Size Infomation

Gating Range 30 - 150 nm

All Events	8331	
Gating Events	6695	
% of all	80.36	
Median	93.2	nm
Mean	99.2	nm
Std Dev.	23.2	nm

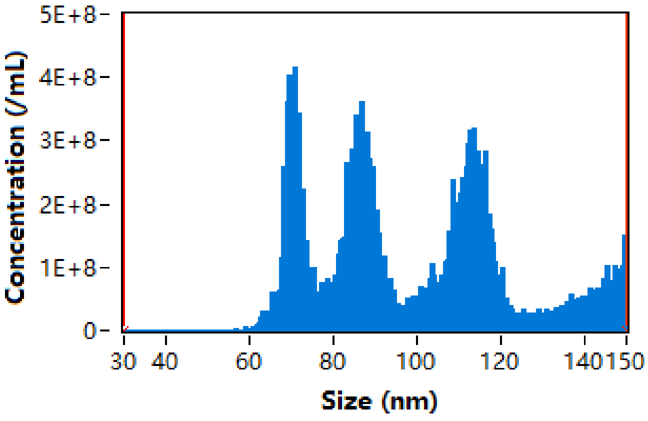

Size (nm)	SS-H
68	143.7
91	416
113	1595.2
155	7855.8
0	0
0	0

Standard Curve   Fit type: Power

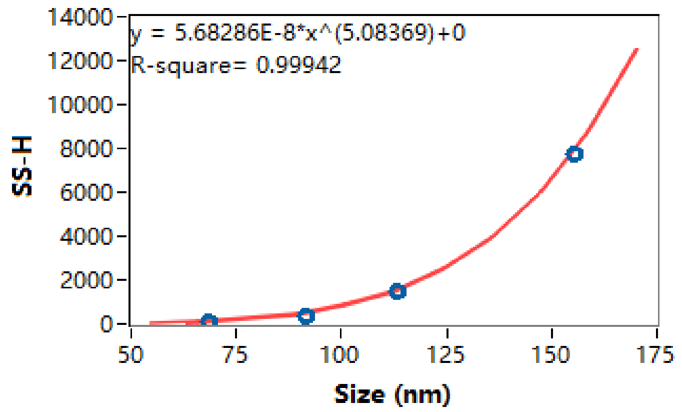

Report By :

2025/11/5 9:50
